# Making your raw data available to the macromolecular crystallography community

**DOI:** 10.1107/S2053230X23007987

**Published:** 2023-09-29

**Authors:** Loes M. J. Kroon-Batenburg

**Affiliations:** a Department of Chemistry, Structural Biochemistry, Bijvoet Center for Biomolecular Research, Faculty of Science, Utrecht University, Utrecht, The Netherlands; Centro Nacional de Biotecnología – CSIC, Spain

**Keywords:** raw data deposition, Zenodo, FAIR principles

## Abstract

A recent editorial in the IUCr macromolecular crystallography journals [Helliwell *et al.* (2019), *Acta Cryst.* D**75**, 455–457] called for the implementation of the FAIR data principles. This implies that the authors of a paper that describes research on a macromolecular structure should make their raw diffraction data available. Authors are already used to submitting the derived data (coordinates) and the processed data (structure factors, merged or unmerged) to the PDB, but may still be uncomfortable with making the raw diffraction images available. In this paper, some guidelines and instructions on depositing raw data to Zenodo are given.

## Abstract 2

Guidance is provided on depositing raw diffraction data to support the results described in a macromolecular crystallography paper.

## 1. Introduction

Advancement in science depends on the reproducibility of scientific results and therefore the sharing of research data is essential (Helliwell *et al.*, 2017 ▸). Open-science models aim at making research data available to the larger community. Open-science platforms have been established, for example the OpenAIRE project (https://www.openaire.eu) and the European Open Science Cloud (EOSC; Jones, 2015 ▸), promoting the sharing of data. Guidelines for proper data management are described in *The FAIR Guiding Principles for scientific data management and stewardship* (Wilkinson *et al.*, 2016 ▸), which requires research data to be Findable, Accessible, Interoperable and Reusable.

A recent editorial in the IUCr macromolecular crystallography journals (Helliwell *et al.*, 2019 ▸) called for the implementation of the FAIR data principles. Authors are encouraged to make their deposited original raw diffraction data publicly available when they submit an article describing a new structure or a new method tested on unpublished diffraction data. In addition, the availability of raw data will allow new science by reanalysis using enhanced methods and technology.

In a series of papers in *Acta Crystallographica Section D* (Terwilliger, 2014 ▸; Kroon-Batenburg & Helliwell, 2014 ▸; Guss & McMahon, 2014 ▸; Terwilliger & Bricogne, 2014 ▸), the possibilities and problems of archiving raw diffraction images have been discussed. Kroon-Batenburg & Helliwell (2014 ▸) estimated the costs of archiving raw diffraction images and brought forward the challenges in reprocessing the data as well as the need to capture and archive the metadata associated with the raw image data. Initially, the costs of raw data storage seemed to be prohibitive. An early initiative of raw crystallographic data archiving, the Store.Synchrotron service of the Australian Synchrotron (Meyer *et al.*, 2014 ▸), paved the way. After this, two large-scale repositories dedicated to diffraction proved that raw data archiving is feasible: the Integrated Resource for Reproducibility in Macromolecular Crystallography (IRRMC) currently has over 9600 data sets (Grabowski *et al.*, 2016 ▸) and the Structural Biology Grid Consortium (SBGrid) currently has 791 data sets (Meyer *et al.*, 2016 ▸). These are most frequently used by high-throughput genomics projects. Also notable is a Polish repository for macromolecular crystallography, MX-RDR (https://icm.edu.pl), that has 402 data sets and rich metadata. The ideal model was set up by the PDBj in 2021 by creating XRDa: an Xtal Raw Data Archive, which aims to collect raw crystal diffraction data for entries submitted to the PDB (Bekker *et al.*, 2022 ▸; https://xrda.pdbj.org/). It provides a complete data record consistent with the FAIR principles.

Large-scale facilities are developing their own data policies. 70% of facilities have a specific clause guaranteeing the long-term preservation of primary data from photon and neutron sources, which is often automatically released to the public after an embargo period during which access is privileged to the original researchers (Götz *et al.*, 2021 ▸). However, with the highly brilliant sources of fourth-generation synchrotrons and X-ray free-electron lasers, as well as improved detector technologies, the data rates increase rapidly such that full data archiving may become impossible. Some raw data sets from XFEL serial crystallography and X-ray coherent imaging experiments can be found in the Coherent X-ray Imaging Data Bank (CXIDB) at https://cxidb.org/ (currently 215 entries). Also noteworthy is EMPIAR, the electron microscopy public archive, that stores raw images underpinning 3D cryo-EM maps and tomograms (https://www.ebi.ac.uk/empiar/) and currently contains 1426 entries and a total of 3.23 PB of storage.

Although raw data reanalysis is not (yet) carried out so frequently, we would like to give some examples of its usefulness. In a study on the effects of dimethyl sulfoxide on the binding of cisplatin and carboplatin to histidine, we used *EVAL* (Schreurs *et al.*, 2010 ▸) to re­analyze the data from 11 different lysozyme crystals that were originally processed with *MOSFLM* (Leslie, 2006 ▸). We studied the possible effects of equipment and data processing on the calculated occupancies and *B* factors of the bound platinum compounds (Tanley *et al.*, 2013 ▸). Using *EVAL*, we also reprocessed some of the data sets for 11 crystal structures of variants of the *Escherichia coli* enzyme *N*-acetylneuraminic lyase as they exhibited twinning and incommensurate modulation (Campeotto *et al.*, 2018 ▸). The reanalysis contributed to understanding why some of the structures were modulated. In the CommDat Workshop *Data Science Skills in Publishing* at ECM32 in Vienna (https://www.iucr.org/__data/assets/pdf_file/0018/144009/07_KroonBatenburg_Rawdata.pdf) we gave two examples of indexing problems in raw data sets, one from SBGrid due to pseudo-merohedral twinning and one from IRRMC that had a second fragment and overlapping reflections.

The workflow of many crystallographers involves collecting data on a home source or a synchrotron facility. With the auto-processing steps implemented on most beamlines, users have no need to download the data to a local computer but can simply use the resulting processed data in their research. The raw data will still be accessible if the facility has proper archiving and data-management systems in place. However, good documentation in a scholarly publication requires the research results to be supported by the original data.

Zenodo is an online repository that was developed and is hosted by CERN and OpenAire, with the support of the European Commission, and allows researchers to share publications and data facilitating open science. It is a highly sustainable solution to data archiving, as the data will be stored for as long as CERN exists. Each data set is assigned a digital object identifier (DOI). Uploads can be made available online immediately. Although Zenodo is a general repository, communities can be established that group together certain types of data. Notably, there are the Macromolecular Crystallography (228 data sets) and Chemical Crystallography (44 data sets) communities.

The authors of papers describing research on a macromolecular structure are already used to submitting the derived data (coordinates) and the processed data (structure factors, merged or unmerged) to the PDB (via *OneDep* at https://www.wwpdb.org/), but may still be uncomfortable with making the raw diffraction images available. Below, we give some guidelines and instructions for depositing raw data to Zenodo.

## 2. How to

**Figure 1:**
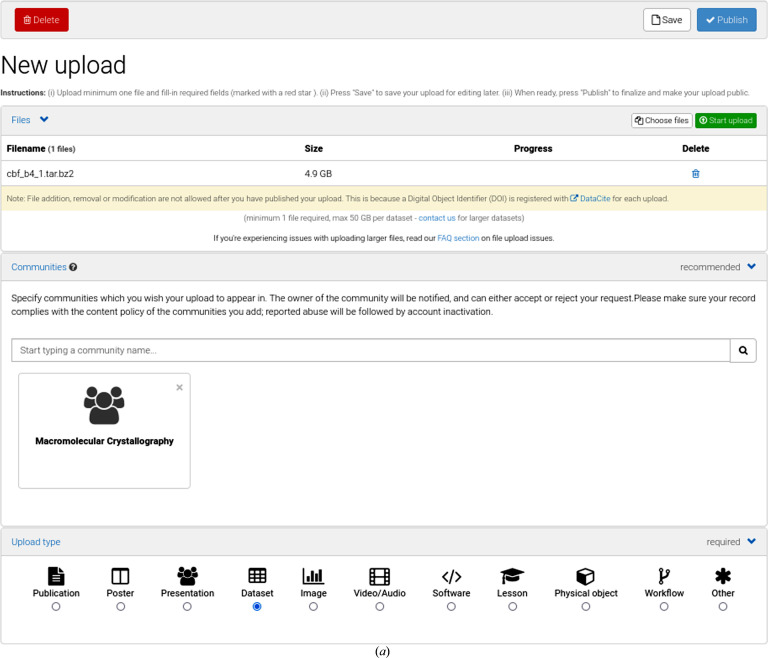

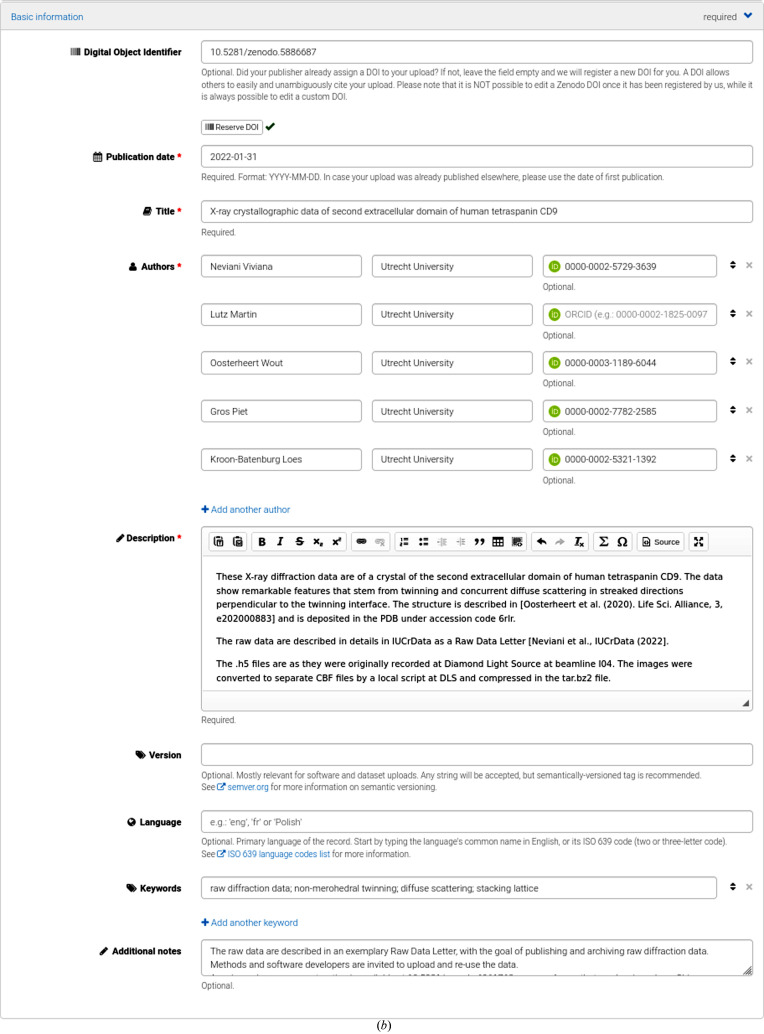

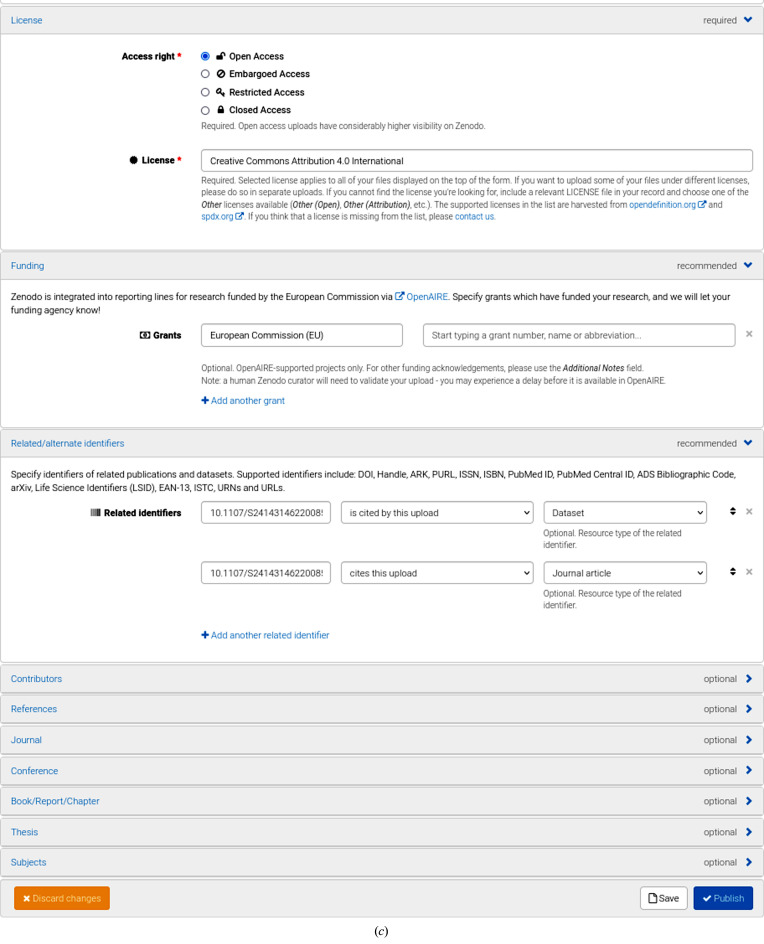
Upload page on Zenodo with the data items as filled in for the first *Raw Data Letter* (Neviani *et al.*, 2022 ▸). (*a*) The raw data file chosen will be uploaded after pressing the ‘Start upload’ button. The Macromolecular Crystallography community has been selected; the upload type is ‘Dataset’. (*b*) ‘Reserve DOI’ resulted in the DOI displayed. The publication date, title and authors and a description of the data set were filled in. Keywords and additional notes were given (see Fig. 2 ▸ for how this will appear on the Zenodo landing page). (*c*) Select ‘Open Access’ and select your preferred license (CC BY 4.0 is the default) while keeping in mind the FAIR data principles. For this data set two related identifiers were given: the imgCIF file describing the metadata and the *Raw Data Letter* as published in *IUCrData*. At the time of uploading the data, the *Raw Data Letter* was not yet published. These identifiers were added at a later stage. Pushing the ‘Publish’ button will result in registration of the data with DataCite. The data can no longer be changed. A new version can be created, however, with the same concept DOI.

**Figure 2:**
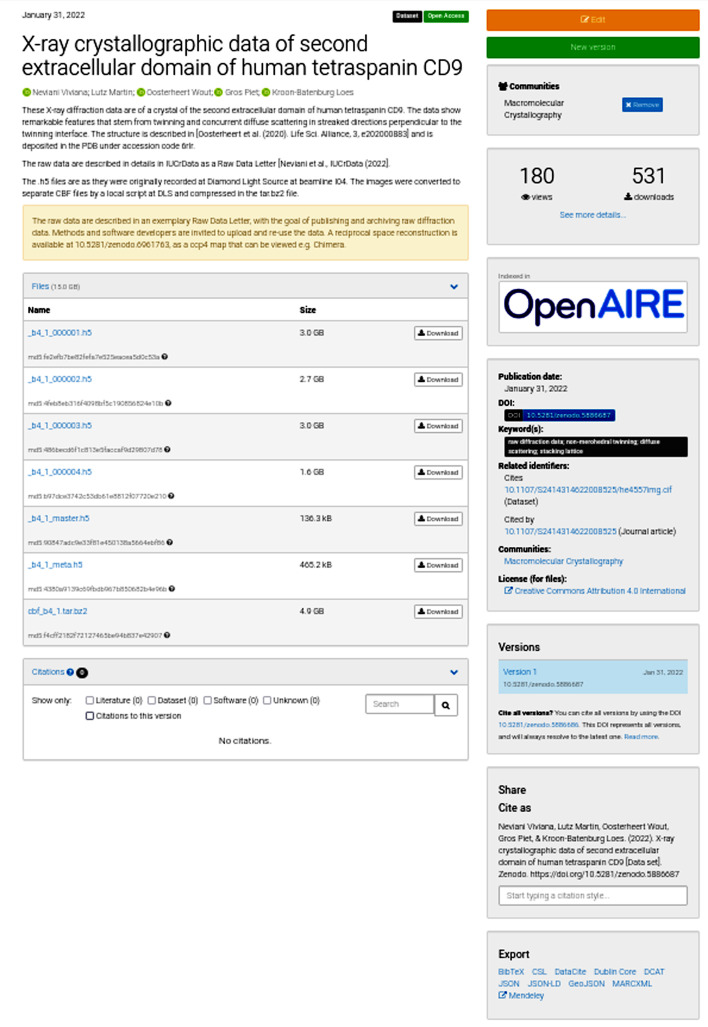
Zenodo landing page for the raw data of the first *Raw Data Letter* (Neviani *et al.*, 2022 ▸). Data are published in the original HDF5 format as well as those converted to CBF.

Zenodo (https://zenodo.org) is a general repository collecting publications, software, data sets, presentations, tutorials and some other types of data. To remove any barriers to publishing raw data, we give some simple instructions for depositing data with Zenodo. To be able to upload data one must first sign up. Once logged in, an ‘Upload’ button is available. The author’s previous uploads are shown, and by clicking ‘New upload’ a questionnaire follows. Choose the data file(s) on your computer and press the ‘Start upload’ button. Any file type is acceptable in Zenodo, although the use of a community standard is recommended (see below for what the preferred data formats may be). The maximum size of a data set is 50 GB. It is wise and convenient for data transfer if you pack your files as zip files or as a tar file compressed using gzip or bzip2. We recommended that you select a suitable community for the data, such as Macromolecular Crystallography. Click ‘Dataset’ as upload type, select ‘Reserve DOI’ and give the title and author details and provide a short description of the data, keywords and additional notes (see Fig. 1 ▸ for the steps in the Zenodo questionnaire). The DOI is registered with DataCite. The metadata will be exported to DataCite and will be searchable. By reserving a DOI, but not publishing, the future DOI can immediately be included in other materials, for example a draft publication or presentations. Also, the raw data content of the data publication can be freely adjusted. Once the ‘Publish’ button has been pressed the data files can no longer be changed. If you need to change the raw data after publication, a new version is created and the original DOI (called the ‘concept DOI’) will point to the most recent version. Additions or updates to the metadata collected by Zenodo (including the overall description) do not result in a new version and so can be freely changed. Related identifiers, such as your IUCr publication, can be given and will appear on the Zenodo landing page (see Fig. 2 ▸ for the resulting landing page for the example data in Fig. 1 ▸). If your paper is not yet published at the time of uploading the raw data, these identifiers can be added later. Although you can restrict access to your data, ‘Open Access’ is the only choice that is compatible with the FAIR principles. Zenodo suggests a CC BY 4.0 license for your data, but you can choose differently. Metadata may be freely reused under the CC0 waiver. Zenodo has Open APIs that allow software to freely access the metadata content (via OAI-PMH) and to deposit large amounts of data.

Raw diffraction data in macromolecular crystallography can come in many different data formats and with varying metadata quality. To ensure reusability, metadata should be accurate and complete or at least sufficient to be able to process the data. Large-scale facilities and diffraction-equipment manufacturers are becoming more and more aware of the FAIR data principles and the metadata content is thus increasing in quality. Recently, a gold standard was proposed by the HDRMX working group (Bernstein *et al.*, 2020 ▸). Noteworthy here are *Raw Data Letters*, which are a collaborative innovation of IUCr Journals with the IUCr Committee on Data, and are part of the journal *IUCrData* (Kroon-Batenburg *et al.*, 2022 ▸). *Raw Data Letters* are meant to describe interesting features in raw data sets that could be of interest to methods and software developers for purposes such as reanalysis by newer methods, or that may be relevant to the structural interpretation. The working group behind *Raw Data Letters* has developed methods to capture the metadata in imgCIF format (Bernstein & Hammersley, 2005 ▸; Hammersley *et al.*, 2005 ▸) by reading the raw images or container files and asking for additional metadata details from the authors. The preferred data standards for *Raw Data Letters* are Nexus/HDF5 and imgCIF/CBF (Kroon-Batenburg *et al.*, 2022 ▸).

## 3. Discussion

We highly recommend that authors make their raw data public so that the crystallographic community will move towards open science. Although in this paper we focus on Zenodo for raw data archiving, the macromolecular crystallography-specific SBGrid (https://sbgrid.org) and IRRMC (https://proteindiffraction.org) could be good alternatives. They both require an associated structure that is deposited in the PDB and both are open to the structural biology community. Some additional metadata are captured at the time of deposition, such as author names, affiliations, compound name, facility/beamline and additional processing details. SBGrid runs the data through an automatic processing pipeline which, when successful, shows the space group, unit cell and some statistics. Success indicates that the metadata are correct and sufficient. Failure is often related to an incorrect beam center. IRRMC provides a diffraction image for each scan, which is very useful if one is searching for data with specific features such as diffuse scattering. We believe that Zenodo is the most sustainable solution to data archiving, and authors can provide as much metadata and related information as they like. Finally, we would like to add that when using the *OneDep* system for structure deposition, authors can provide a DOI for their associated raw data, so linking the raw data to a PDB entry is straightforward.
